# Diagnostic Model of Superficial Lymph Nodes Based on Clinical History and Ultrasound Findings: A Prospective Cohort Study

**DOI:** 10.3389/fonc.2021.756878

**Published:** 2022-01-03

**Authors:** Xiao-Qu Tan, Lin-Xue Qian, Jun-Feng Zhao, Peng-Fei Sun, Qing-Qing Li, Ruo-Xuan Feng

**Affiliations:** Department of Ultrasound, Beijing Friendship Hospital, Capital Medical University, Beijing, China

**Keywords:** ultrasound, lymph node, S/L ratio, medical history, diagnostic model

## Abstract

**Objectives:**

Differentiation of benign and malignant changes in lymph nodes is extremely important. We aimed to identify the ultrasound and clinical diagnostic criteria permitting this differentiation.

**Methods:**

Clinical and ultrasound data were collected at Beijing Friendship Hospital from May 2019 to November 2020. Univariate and multivariate analysis were performed using statistical methods, and a mathematical model was established to evaluate benign and malignant lymph nodes.

**Results:**

A total of 1343 LNs (person) with US‐guided core needle or fine needle biopsy (CNB or FNB) were evaluated in the analysis. Variables with a high predictive power were sex (odds ratio, OR: 3.360, *p*<0.001), short diameter (OR: 4.660, *p*<0.001), short/long diameter (S/L) ratio (OR: 1.515, P=0.007), border (OR: 1.626, *p*=0.002), cortex echogenicity (OR: 2.089, P<0.001), fusion (OR: 2.313, *p*=0.002), vascularity (peripheral vascularity, OR: 3.424, *p*<0.001; mixed vascularity, OR: 4.127, *p*<0.001), and medical history (fever/local pain, OR: 0.316, *p*<0.001; tumor history in the drainage area, OR: 4.595, *p*<0.001; both, OR: 5.554, *p*<0.001). The cut-off score on receiver operating characteristic (ROC) curve analysis using these eight variables was 2.5. The largest area under the ROC curve (Az) value was 82.3% (95% confidence interval (CI), 0.805–0.851), and the sensitivity (79.4%), specificity (72.3%), and accuracy (74.8%) were higher than those for nearly all the single indices.

**Conclusion:**

The model of combination of ultrasound and clinical symptoms can preliminarily evaluate the benign and malignant of lymph nodes.

## Introduction

Lymph nodes (LNs) are an important part of the human immune system. The human body contains approximately 800 LNs (1), and more than one-third of these are located in superficial areas. LNs participate in the immune process by filtering out the lymph. In organs or regions showing pathological changes, bacteria, toxins, parasites, or cancer cells can enter the corresponding local LNs. LNs clear or block harmful factors by proliferating, increasing in volume, or showing changes in shape and architecture. The lymphatic system itself can also show pathological changes. Malignant cells in the LNs proliferate and multiply, which can squeeze the normal structures and increase the volume of LNs. Thus, identification and characterization of benign and malignant changes in LNs is of great clinical significance.

High-frequency ultrasound (US) is a real-time, convenient, cheap, and non-invasive technique compared with computed tomography (CT) and positron emission tomography (PET)/CT that has recently gained popularity. The US features of LNs, including increases in LN volume, shape changes, fuzzy boundaries, internal calcification, and necrosis, have been found to show some diagnostic value in differentiating benign and malignant LNs (2–4). However, the specificity of single parameters is low, and the same US features can appear in different diseases, e.g., a round shape can appear in both tuberculous LNs and lymphoma (5, 6). Moreover, US features can vary across different stages of the same disease. For example, an echogenic hilum may appear in the early stage of LN metastasis and disappear in the late stage (7). Therefore, evaluations based on changes in single characteristics cannot serve as diagnostic criteria for LN disease, and comprehensive diagnostic criteria, including medical history and US features, are required.

## Materials And Methods

### Participants

This study was conducted at Beijing Friendship Hospital from May 2019 to November 2020 and included the data for superficial LNs assessed using fine or coarse needle biopsy. All patients provided informed consent. We included patients who agreed to undergo US-guided biopsy of LNs, had LNs that could be visualized using US, and those who signed the informed consent form. Only one lymph node per patient was selected. The exclusion criteria were as follows: (i) poor coagulation function, small LNs, or LNs located at sites not suitable for biopsy, (ii) unclear for histological or cytological diagnosis. The classification of the pathological results was based on Jaffe’s book (8). The benign LNs mainly showed reactive lymphadenopathy, while malignant lesions included cases of lymphoma and LN metastasis. A total of 1416 LNs were collected, of which 73 were not confirmed by final pathological diagnosis, and the remaining 1343 LNs (person) entered the final study.

### Instrument

Sonographic examinations and US-guided fine or coarse needle biopsy was performed using Hi Vision Ascendus (Hitachi, Kashiwa, Chiba, Japan) US units and a high-frequency (7.5–12 MHz) linear-array transducer.

### Examination and Operation Protocol

Before the puncture, the patients’ age, sex, past and current medical history, and chief complaints were recorded. The patient was asked to remain supine, exposing the neck, armpit, or groin. The relevant areas were scanned by US to identify LNs that needed to be punctured. The long and short diameters were measured on the section showing the maximum long diameter, and the image was saved. Special features such as cystic changes and calcification were evaluated, and the respective images were also saved. We also saved the colour Doppler flower images and images of the peripheral and contralateral LNs.

US‐guided core needle or fine needle biopsy (CNB or FNB) was performed by a physician with more than five years of experience in interventional US. The core needle was an 18‐gauge, double-action, spring‐activated needle (1.1-cm excursion; TSK Ace‐cut; Create Medic, Yokohama, Japan), and the fine needle was a 25-gauge needle (BD biosciences, NJ, USA). The biopsy needle tip was manually advanced to the target LN by using a free‐hand technique under US guidance. For CNB, two biopsy samplings of 1 cm or 2 cm were performed, and the specimens were stored immediately in 10% neutral-buffered formalin solution. For FNB, the aspirated materials were placed on labelled glass slides, smeared, and fixed in 95% ethyl alcohol. All specimens were fixed and stained according to the standard protocol for histologic or cytological examinations. The patients were monitored for an additional 30 min for potential complications.

### Data Arrangement

The pathological findings of LNs and the patients’ final diagnoses were followed-up three months after CNB or FNB. Data for sex, age, fever or local pain, and history of tumor in the drainage area were obtained and recorded in the form. In addition to a previous tumor history, if the tumor in the drainage area of the LN was found before the puncture date and confirmed to be malignant by surgery or biopsy, the answer to the tumor history question was marked as ‘yes’. US image features were observed by two double-blinded sonographers with more than 10 years’ experience, who subsequently completed the form. The US image features included long diameter, short diameter, short/long diameter (S/L) ratio, border (sharp, blurry), margin (regular, irregular), echogenic hilum (present, absent), echogenicity of the cortex (homogeneous hypoechoic cortex, inhomogeneous cortical echo, including local enhancement or reduction and necrosis), calcification (present, absent), vascularity (avascular, hilar vascularity, peripheral vascularity, and mixed vascularity), single/multiple, fusion (yes, no), and laterality (unilateral, bilateral).

### Statistical Analysis

All the statistical analyses were performed using SPSS version 19.0 (SPSS, Inc., IL, USA). Interobserver agreement analysis was performed by randomly selecting a group of data points for analysis. Chi-square test was used for counting data. Brown-Forsythe test was used to test the homogeneity of variance of measurement data, and an independent t-test or one-way ANOVA was used according to the test results. A receiver operating characteristic (ROC) curve analysis was performed to determine the cut-off values showing statistical significance for the measurement data (*p* ≤ 0.05). Binary logistic regression was used to identify independent predictive variables. Variables with a high predictive power (*p*< 0.01) were selected and scored. Cross-validation was used for the variables in question. Lastly, an ROC curve analysis was applied again to identify the cut-off value for the total LN score. Graphpad Prism version 9.0 was used to convert statistical results to graphics.

## Results

### Primary Findings

A total of 1343 LNs (person) were evaluated in the analysis. As shown in **Table 1**, benign LNs mainly included non-specific LN reactive hyperplasia (84.8%), granulomatous lymphadenitis (8.8%), and Kikuchi’s disease (5.3%). Malignant LNs included lymphoma (19.3%) and LN metastasis (80.7%). Most of these LNs (75.7%) were located in the neck.

**Table 1:** Types and locations of benign and malignant lymph nodes.

	Benign LNs (n = 871)	Malignant LNs (n = 472)
pathological	non-specific reactive lymphadenopathy 739	lymphoma 91
classificaton	granulomatous changes 77	metastatic cancer 381
	Kikuchi's diaease 46	thyroid origin 144
	infectious mononucleosis 4	breast origin 84
	changes related to autoimmune diseases 2	lung origin 76
	epstein barr virus infection 1	digestive tract origin 19
	Castleman disease 1	naopharynx and oral origin 8
	hemophagocytic syndrome 1	muliebria origin 10
		urothelium of urinary system origin 4
		prostate origin 7
		mediastinum origin 2
		unkown origin 27
position		
neck	656	361
I	10	0
II	159	44
III	161	76
IV	299	226
V	16	11
VI	11	4
armpit	155	88
groin	60	23

### Comparative Analysis of Benign and Malignant LNs

**Figure 1:**
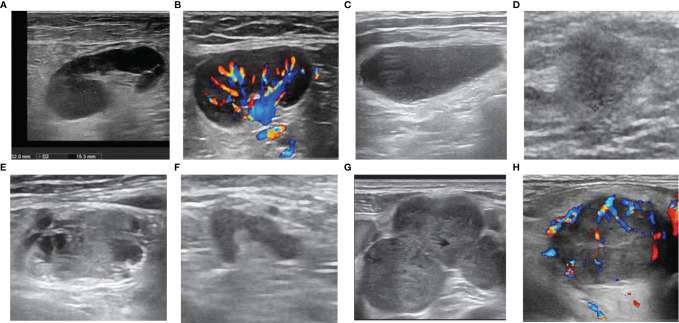
Ultrasonographic features of lymph nodes (LNs). LNs with **(A)** sharp border, regular margin, hilum, homogeneous cortex, **(B)** hilar vascularity, **(C)** absence of hilum, **(D)** blurred border, **(E)** calcification and inhomogeneous cortex (necrosis), **(F)** irregular margin, **(G)** fusion, **(H)** peripheral vascularity.

As shown in **Table 2**, the interobserver agreement was relatively high (κ > 0.80). The presence of single/multiple LNs was not significantly different (*p*= 0.886). Malignant LNs were commonly seen in men (*p* < 0.01), those with a tumor history in the drainage area (*p*< 0.01), and those with no history of fever or local pain (*p* < 0.01). On the US images, the features characterising malignant LNs (**Figure 1**) were a blurry border (*p* < 0.01), an irregular margin (*p* < 0.01), disappearance of an echogenic hilum (*p* < 0.01), inhomogeneous cortical echo (*p* < 0.01), calcification (P < 0.01), peripheral or mixed vascularity (*p* < 0.01), fusion (*p* < 0.01), and unilateral presentation (*p*< 0.01). The patients with malignant LNs were older (*p*< 0.01) and the malignant LNs were larger (long diameter and short diameter, *p* < 0.01) and more round (S/L value was larger, *p* < 0.01). In the ROC curve analysis, the optimal cut-off values were 55.5 years for age (the largest area under the ROC value [Az] = 0.602, 95% confidence interval [CI]: 0.570–0.633), 1.85 cm for the long diameter (Az = 0.595, 95% CI: 0.563–0.626), 0.75 cm for the short diameter (Az = 0.712, 95% CI: 0.683–0.741), and 0.46 for the S/L ratio (Az = 0.660, 95% CI: 0.630–0.690).

**Table 2:** Univariate analysis of benign and malignant lymph nodes.

	κ value	Benign LNs (n = 871)	Malignant LNs (n = 472)	p value
**people**				
sex(male/female)	–	292(33.5%)/579(66.5%)	203(43.0%)/269(57.0%)	<0.001
age(year)	–	48.15±16.32	54.06±16.00	<0.001
fever or local pain(yes/no)	–	190(21.8%)/681(78.2%)	39(8.3%)/433(91.7%)	<0.001
tumor history(yes/no)	–	340(39.0%)/531(71.0%)	316(66.9%)/156(33.1%)	<0.001
**US features**				<0.001
long diameter(cm)	–	1.82±0.96	2.17±1.13	<0.001
short diameter(cm)	–	0.76±0.48	1.14±0.62	<0.001
S/L ratio	–	0.46±0.19	0.55±0.18	<0.001
border(sharp/blurred)	0.83	718(82.4%)/153(17.6%)	291(61.7%)/181(38.3%)	<0.001
margin(regular/irregular)	0.89	757(86.9%)/114(13.1%)	361(76.5%)/111(23.5%)	<0.001
echogenic hilum(exist/disappear)	0.92	520(59.7%)/351(40.3%)	380(80.5%)/92(19.5%)	<0.001
echogenicity of the cortex(homogeneous/inhomogeneous)	0.95	677(77.7%)/194(22.3%)	237(50.2%)/235(49.8%)	<0.001
calcification(present/absent)	0.90	103(11.8%)/768(82.2%)	121(25.6%)/351(74.4%)	<0.001
vascular	0.92			<0.001
hilar	–	470 (54.0%)	130 (27.5%)	
avascular	–	285 (32.7%)	124 (26.3%)	
mixed	–	42 (4.8%)	84 (17.8%)	
peripheral	–	74 (8.5%)	134 (28.4%)	
multiple/single	–	699(80.3%)/172(19.7%)	375(79.4%)/97(20.6%)	0.886
fusion(yes/no)	0.86	37(4.2%)/834(95.8%)	86(18.2%)/386(81.8%)	<0.001
lateral(bilateral/unilateral)	–	356(40.9%)/515(59.1%)	144(30.5%)/328(69.5%)	<0.001

**Figure 2:**
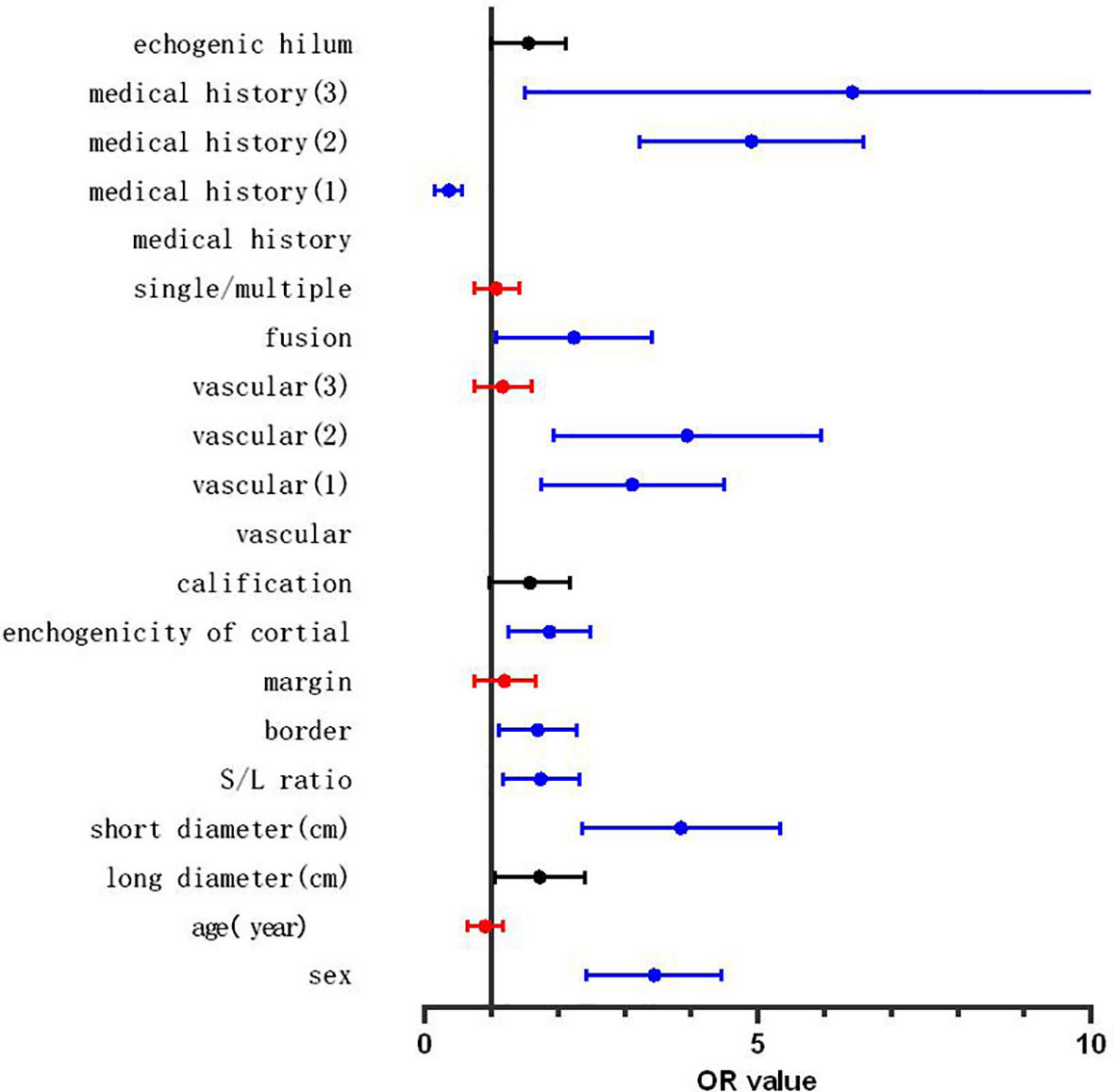
Forest map of the results of binary logistic regression. Red line: the variables with no independent predictive power (*p* > 0.05), blue line: the variables showing significant independent predictive power (*p* < 0.01), black line: the variables showing a certain independent predictive power (0.05 > *p* > 0.01).

These statistically significant indicators were entered into the binary logistic regression analysis. Pain or local pain and tumor history in the drainage area were incorporated into medical history. The definitions of the statistics are shown in **Table 3**, and the results are shown in **Figure 2**. The variables with a high predictive power (*p* < 0.01) were sex (odds ratio [OR]: 3.360, 95% CI: 2.506–4.505, *p* < 0.001), short diameter (OR: 4.660, 95% CI: 3.359–6.465, *p* < 0.001), S/L ratio (OR: 1.515, 95% CI: 1.122–2.045, *p* = 0.007), border (OR: 1.626, 95% CI: 1.153–2.293, *p* = 0.002), cortex echogenicity (OR: 2.089, 95% CI: 1.522–2.869, *p* < 0.001), fusion (OR: 2.313, 95% CI: 1.365–3.918, *p* = 0.002), vascularity (peripheral vascularity, OR: 3.424, 95% CI: 2.221–5.278, *p* < 0.001; mixed vascularity, OR: 4.127, 95% CI: 2.472–6.890), and medical history (fever or local pain, OR: 0.316, 95% CI: 0.179–0.560, *p* < 0.001; tumor history in the drainage area, OR: 4.595, 95% CI: 3.272–6.452, *p* < 0.001; and both, OR: 5.554, 95% CI: 2.43–12.667, *p* < 0.001). The overall prediction accuracy was 80.6%, while the prediction accuracy in intra-group random verification (random 70% data) was 81.0%. The interaction between echogenic hilum and size (long diameter and short diameter) was analysed and the variables showed no interaction (echogenic hilum and long diameter: *p*= 0.328; echogenic hilum and short diameter: *p*= 0.386).

**Table 3:** The definition of variables in logistic regression.

Variables	Definition
sex	male=0, famale=1
age(year)	“<55”=0,"≥56"=1
medical history	no history=0, fever or local pain=1,
	tumor history in drainage area=2, both of them=3
long diameter(cm)	“<1.9”=0,“≥1.9”=1
short diameter(cm)	“<0.8”=0,“≥0.8”=1
S/L ratio	“<0.5”=0,“≥0.5”=1
border	sharp=0,blurry=1
margin	regular=0, irregular=1
echogenic hilum	exist=0,disappear=1
echogenicity of the cortex	homogeneous=0, inhomogeneous=1
calcification	invisible=0, visible=1
vascular	hilar vascular=0, peripheral vascular=1,
	mixed vascular=2, avascular=3
fusion	no=0, yes=1
lateral	bilateral=0, unilateral=1

### Establishment of the Mathematical Model

**Figure 3:**
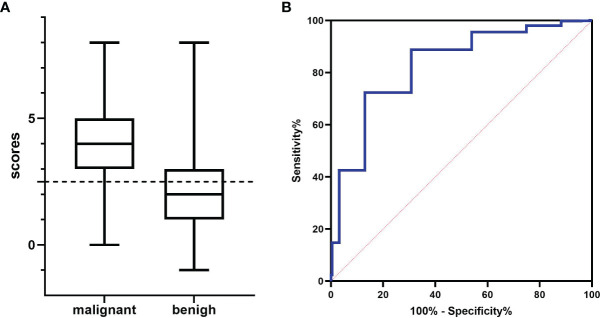
The results for various data scores. **(A)** Histogram analysis: the cut-off value was 2.5 (dotted line). **(B)** Receiver operating characteristic (ROC) curve analysis: the largest area under the ROC curve (Az) value was 82.3%.

Eight of the variables described above were scored as follows: sex (female = 0, male = 1), medical history (no history = 0, fever or local pain = -1, tumor history in the draining area = 1), short diameter (<0.8 cm = 0, ≥0.8 cm = 1), S/L ratio (<0.5 = 0, ≥0.5 = 1), border (sharp = 0, blurry = 1), cortex echogenicity (homogeneous hypoechoic cortex = 0, inhomogeneous cortical echo = 1), vascularity (hilar vascularity or avascular = 0, peripheral vascularity or mixed vascularity = 1), and fusion (no = 0, yes = 1). The cut-off score was 2.5 on the ROC curve analysis (**Figure 3**). Therefore, the diagnostic criteria for malignant LNs were the presence of ≥3 variables (≥4 variables in patients with only fever or local pain). The largest area under the ROC curve (Az) value was 82.3%; 95% CI was 0.805-0.851; and sensitivity, specificity, and accuracy were 79.4%, 72.3%, and 74.8%, respectively, which were higher than those for almost all of the single indexes (**Table 4**).

**Table 4:** Sensitivity, specificity, and accuracy of variables in the model.

Variables	Sensitivity(%)	Specificity(%)	Accuracy(%)
Sex	43.0	66.5	58.2
medical history	66.9	61.0	63.1
short diameter(cm)	70.3	61.9	64.9
S/L ratio	69.5	56.9	61.9
border	38.3	82.8	66.9
echogenicity of the cortex	49.8	77.7	67.9
vascular	46.2	86.7	72.4
fusion	18.2	95.8	68.5

## 4 Discussion

A mature standardized US imaging reporting system was established for imaging of the breast, thyroid, and other sites (9, 10). Kyeong (3) tried to establish a 5-point scale system with 291 LNs in 2015. The variables in their regression equation were shape, echogenicity, echogenic hilum, calcification, vascularity, and real-time elastography assessment findings, and the probability of malignant LNS with ≥3 variables was 5.9–99.8%. We established a new prediction model by selecting 8 of 16 variables representing population information and US features.

Clinical variables were included in our study. We found that LNs with the same US features but different medical histories had different final pathological diagnoses. Thus, medical history was shown to be an independent predictor with the highest odds ratio among all the parameters.

Another difference from previous studies was in relation to the echogenic hilum (2–4). The echogenic hilum is primarily composed of fat tissue, blood and lymph vessels, and connective tissue, and it is hyperechoic due to the presence of many acoustic boundary surfaces (8). The rich cellularity (B and T lymphocytes) was the cause of the hypoechoic cortex. The rate of occurrence of a hilum has been suggested to be related to the LN size. In the article by Michael et al. (11), the rate of occurrence of a hilum in LNs with short diameter < 5 mm was 37.9–79.7%. We verified the interaction between the hilum and the size parameters (long and short diameters) and found no interaction between them. This result showed that the presence of the hilum was not affected by LN size. However, our results may have been affected by selection bias. All of our enrolled LNs were from biopsy specimens, and LNs without a hilum were more likely to be selected. The hilum was absent in 520 (59.7%) benign LN specimens and 380 (80.5%) malignant LN specimens. Pathologically, the initial LNs were LNs with only primary lymph follicles in the cortex and no antigen stimulation and were barely observable by US. The benign changes in LNs were primarily reactive lymphadenopathies, including follicular hyperplasia, sinus histiocytosis, and paracortical hyperplasia (8). The proliferation caused by antigen stimulation can squeeze the medulla, resulting in the disappearance of the echogenic hilum. However, the relationship between the location of hyperplasia and the disappearance of the echogenic hilum remains to be studied. In our daily clinical practice, we have often observed LNs without a hilum around level VI in patients with Hashimoto thyroiditis. Malignant LNs also showed an echogenic hilum in the early stage (7). In some studies, narrowing of the hilum or local thickening of the cortex (12, 13) were considered as markers of malignant LNs; however, we did not consider this variable because the criteria were difficult to define.

The findings for calcification were also different from those reported previously, since calcification may be related to the type of disease. Previous studies had shown a high incidence of calcification in tuberculosis (5) and thyroid cancer metastasis (14). In our data, granulomatous changes accounted for 8.8% of benign LNs, while metastatic cancer of thyroid origin accounted for 30.5% of malignant LNs, which increased the incidence of calcification in the malignant LN group. Although calcification was not included in the final diagnosis, it can be used as a reference for specific disease differentiation.

The sensitivity and specificity of our final diagnosis model were 79.4% and 72.3%, respectively, which were higher than those of almost all single variables. The results also showed that diagnosis of LNs lacked specific indicators. This may be attributed to the fact that the LN itself is a filtering organ. Benign factors such as bacterial, viral, and fungal infections and malignant factors such as neoplastic infiltration, including haematological disorders, can both cause proliferative and structural changes. These findings also illustrated the importance of medical history. Studies on individual diseases have yielded some specific indicators. For example, metastases from thyroid cancer were characterised by calcification and cystic degeneration (14), and LNs of lymphoma showed reticular echoes (6). In addition, newer techniques such as contrast-enhanced sonographic imaging and elastography may facilitate diagnosis (15). Thus, our findings provide a basic direction for diagnosis of LN diseases.

A limitation of this study was that this was a single-center study, and the findings were affected by the disease types and the lack of external verification.

Finally, malignant tumors can be diagnosed if patients meet ≥3 of the following criteria (≥4 criteria for patients with only fever or local pain): (i) male sex, (ii) history of tumor in the drainage area (with or without fever and local pain), (iii) short diameter ≥ 0.8 cm, (iv) S/L ratio ≥ 0.5, (v) blurred border, (vi) inhomogeneous cortical echo, (vii) peripheral or mixed vascular on colour Doppler flower images, and (viii) fusion of LNs.

## Data Availability Statement

The raw data supporting the conclusions of this article will be made available by the authors, without undue reservation.

## Ethics Statement

This study was approved by the Ethics Committee and Institutional Review Board of Beijing Friendship Hospital (approval number: 2019-P2-093-02). The patients/participants provided their written informed consent to participate in this study. Written informed consent was obtained from the individual(s) for the publication of any potentially identifiable images or data included in this article.

## Author Contributions

XT analyzed the data and was a major contributor in writing the manuscript. JZ, RF, PS, and QL collected data. LQ designed and revised the conception. All authors contributed to the article and approved the submitted version.

## Funding

This investigation was supported by the Medical Image Database Foundation of China, grant number: CSQBLC2019JJSJ001. The funder was not directly involved in this study.

## Conflict of Interest

The authors declare that the research was conducted in the absence of any commercial or financial relationships that could be construed as a potential conflict of interest.

## Publisher’s Note

All claims expressed in this article are solely those of the authors and do not necessarily represent those of their affiliated organizations, or those of the publisher, the editors and the reviewers. Any product that may be evaluated in this article, or claim that may be made by its manufacturer, is not guaranteed or endorsed by the publisher.
